# Sunderland Dispensary

**Published:** 1829-07-01

**Authors:** 


### XXXIX.

### SUNDERLAND DISPENSARY.

#### 1. CASE OF CHOREA, UNDER DR. BROWN.

Jane Wilson, æt. 16, had the ordinary symptoms of chorea in the beginning of January, 1829: she dragged the *left* leg considerably in walking, and had the gesticulations customary in that disease in the arm of the *same* side. The tongue was furred; the bowels were torpid; the complexion was pale and unhealthy; the pulse frequent; there was apparent hebetude of intellect, and she felt giddiness and pain of the head. Several leeches were applied to the temples, and purgatives were prescribed. From circumstances Dr. B. did not see her again till the 29th of the month, when she was brought to the Sunderland Infirmary, in general and violent convulsions, and placed under his care. She was very largely bled from the arm and from the temporal artery; leeches and cold stupes were applied to the head; calomel and other strong purgatives, and subsequently oil of turpentine, both by mouth and in enemata, were administered: and on the sixth of February, the catamenial discharge having appeared, there was a cessation of the convulsive movements, which, till that time, had been almost incessant, and so violent, that the patient had been kept in bed only by being firmly bound to it. Her intellect, which had been almost entirely obscured even in the short intervals of the convulsions, became so far clear that she answered questions correctly; she complained much of pain in the head; the circulation was quick and rather forcible.

The menstrual discharge ceased on the 8th of February; and, on the following day, there was a renewal of all the violent symptoms. The same remedies were adopted but without avail; the poor girl becoming comatose on the night of the eleventh, and dying early in the morning of the twelfth.

*Appearances on dissection.* The whole surface of the brain was unusually vascular. Some *lymphoid* serum was effused between the arachnoid coat and pia mater; the lateral ventricles contained half an ounce of serum; the choroid plexus was unusually vascular; the vena galeni and sinuses of the dura mater were full of blood. But the most remarkable appearance connected with the brain was a calcareous concretion in the medullary substance of the *left* hemisphere. This body was of an irregular cubic form, and each of its sides measured about half an inch.

The thoracic and abdominal viscera were perfectly healthy: there was a small hydatid formation adhering to the right ovarium.

"I beg to remark (says Dr. B.) that this is the third case of fatal chorea which has fallen under my observation; or, at least (that I may obviate a verbal cavil) the third case in which the partial convulsions designated by this term have passed into general convulsions, coma and death; but in the present instance only have I had an opportunity of illustrating the pathology of the disease by examination after death. The result of this examination, the mode in which life is terminated, the symptoms of the disease whilst the convulsions are only partial, and the effects of remedies upon it, lead me to the conclusion, that it arises more frequently from direct irritation of the brain with increased afflux of blood thither, than merely sympathetic excitement of this organ. The bold depletory measures applied by Sydenham to this disease might induce us to believe, that his opinion of its nature was not materially different from that here expressed; and even the indefinite language, in which he endeavours to embody that opinion, reduced into a more precise form conveys very exactly the same idea: "cum adfectus iste ab humore aliquo in nervos irruente, quorum irritatione istius modi motus præternaturales producuntur, pendere mihi videretur, &c." Substitute the word "blood" for a "certain humor;" and suppose the impulse to be to the *origins* of the nerves, and his opinion is very exactly that which I am espousing. The whole passage, whence the extract is made, is an excellent example of the superiority of this distinguished physician's practice to his reasoning, or rather to the language, derived from the Galenical school, in which he has unfortunately cloathed that reasoning. My success in treating the disease by purgatives only, has been by no means so great as that of Drs. Parr and Hamilton; that this plan will frequently succeed I grant; but there are few cases in which bleeding, general or local, may not at the same time be advantageously employed, and some in which it is essentially necessary. When mere purgatives remove the disease, I should attribute it in most cases rather to their relieving the organ primarily affected, than to their withdrawing sympathetic irritation from that organ by expelling noxious matters from the bowels. It may appear somewhat difficult to reconcile this opinion respecting chorea with the acknowledged efficacy of arsenic in its cure, an efficacy which I have often experienced; but if we consider the influence of this mineral over some forms of cutaneous inflammation (lepra vulgaris for instance) and perhaps over ague and intermittent neuralgiæ, the difficulty will vanish."

N.B.—It will have been observed, that the convulsive movements were on the *same side* as the irritating substance in the brain.

### II. Case of Purpura Hæmorrhagica.

Thomas Dixon, ætat. 22, became a patient of the Sunderland Dispensary, on the 10th of March of the present year. He had profuse hæmorrhage from the bowels; his legs and thighs were covered with petechiæ, and there were some on the face; they existed too on the lining of the mouth, and the thin cuticle covering the tongue being ruptured in three points, the blood flowed from his mouth as he lay. His countenance was pale; his pulse 120, small and hard; he had excessive thirst; complained of much pain in the head, and his bowels were rigidly constipated.

He was freely bled from the arm on the 13th, 14th, and 16th, his bowels were opened by pills of calomel, extract of colocynth and croton oil; sulphuric acid and acetate of lead with opium were administered; by which means the hæmorrhage was speedily subdued, and on the 25th of March, all traces of the petechiæ had disappeared.

The blood drawn was the richest in coagulating and colouring matter Dr. B. ever beheld. Even after standing for twenty-four hours, it threw out only a few globules of serum.

Dr. B. has related this case for the purpose of noticing a coincidence which may *perhaps* (should it fall under the observation of others) throw some light on this obscure disease. The subject of the present case has been for years liable to obstructed bowels, with occasional violent pain there and vomiting, requiring the administration of powerful purgatives; and though now all hæmorrhage has ceased even from the bowels, yet there is still disease existing there. The abdomen is tense and very painful on pressure; the pulse is frequent; the tongue white.; the thirst great, and there is either constipation or watery diarrhœa. About a year ago, Dr. B. had an opportunity of examining the body of a female of intemperate habits, who fell a victim to purpura hæmorrhagica. The only morbid appearance discovered (besides the incorporation of blood with the cutis in various parts of the body) was extensive ulceration of the cæcum and right extremity of the colon.

